# In cervical arthroplasty, only prosthesis with flexible biomechanical properties should be used for achieving a near-physiological motion pattern

**DOI:** 10.1186/s13018-020-01908-y

**Published:** 2020-09-09

**Authors:** Manfred Muhlbauer, Ernst Thomasch, Wolfgang Sinz, Siegfried Trattnig, Hermann Steffan

**Affiliations:** 1 Neurosurgical Department, Donauspital SMZ-Ost, Langobardenstrasse 122, 1220 Vienna, Austria; 2 Vehicle Safety Institute, Graz University of Technology, Graz, Austria; 3 High Field MR Center, Department of Biomedical Imaging and Image-guided Therapy, Medical University of Vienna, Vienna, Austria

**Keywords:** Cervical spine biomechanics, Cervical arthroplasty, In vivo kinematic study

## Abstract

**Background:**

In cervical arthroplasty, qualitative motion analysis generally investigates the position of the center of rotation (COR) before and after surgery. But is the pre-op COR suitable as reference? We believe that only a comparison against healthy individuals can answer whether a physiological motion pattern has been achieved. The aim of our study was to examine how the COR for flexion/extension after insertion of 3 biomechanically completely different types of disc prostheses compares to healthy volunteers, and whether and how prosthesis design contributes to a more natural or maybe even worse motion pattern.

**Methods:**

In 15 healthy volunteers, MRI in flexion and in extension was taken, and the coordinates for the CORs (COR-HV) from C3 to C7 were determined. Then pre- and post-op flexion/extension x-rays from 30 patients with a one-level disc prosthesis underwent analysis for determination of COR from C3 to C7; 10 patients who received a Bryan, a Prestige STLP, or a Discover prosthesis were chosen, respectively. Change of post-op COR position was investigated in relation to the COR-HV.

**Results:**

The pre-operative COR is not congruent with the COR found in healthy subjects and therefore cannot be used as reference for investigation whether a disc prosthesis resembles natural motion. However, the comparison with healthy individuals shows that prosthesis insertion can change the coordinates of the COR to any direction in all levels from C3/4 to C6/7 regardless of the operated segment. Prostheses with flexible biomechanical properties can contribute to shift the COR toward normal, but devices with unphysiological biomechanical design, like fixed ball socket designs, for instance, can make the motion pattern even worse.

**Conclusions:**

Even if the small cohorts in our study do not allow strong conclusions, it seems that in cervical arthroplasty, the biomechanical concept of the prosthesis has a significant impact whether a near-physiological motion pattern can be achieved or not. As it is a rumor but not scientifically proven that prosthesis design has no influence on clinical outcome, surgeons should only choose devices with flexible biomechanical properties for disc replacement.

## Background

Cervical disc prostheses are used to preserve motion after discectomy. But is quantitative motion preservation enough, and is it really unimportant which biomechanical concept they have? The market offers so many different devices, some with a ball socket design but with totally different radii, devices with their COR below or the COR above the respective motion segment, some with modified ball socket design that allows translation, again with their COR below or above the prosthesis, devices with 2 articulating surfaces, and finally devices with no articulating surfaces at all but motion through an elastic nucleus.

It is widely believed that the biomechanical concept has no impact on clinical outcome; however, this is hard to imagine considering the completely different motion patterns of these devices. And in fact, this is rumor and was never verified with a randomized study directly comparing different devices with respect to clinical outcome. Therefore, we believe a cervical disc prosthesis should resemble physiological motion as close as possible, and biomechanical studies on qualitative motion are still important. A variety of studies already investigated what can happen with the COR after insertion of a prosthesis, but they all use the pre-op COR as reference [1–8]. We believe that in patients with disc herniations, the COR of the affected segment is not at a physiological position anymore; therefore, investigation of qualitative motion of a disc prosthesis must compare the post-op COR against healthy volunteers rather than with the pre-op COR. This was the aim of our study, to compare pre- and post-OP COR for maximum flexion/extension from 30 patients with 3 different types of disc prostheses with 15 healthy volunteers in whom the COR was determined from flexion/extension MRI.

## Materials and methods

### Healthy volunteers

Fifteen healthy volunteers (6 males, 9 females; age 25–53 years; mean age 37.5 years) with no previous symptoms of cervical spondylosis underwent MRI-investigation of their cervical spines after giving informed consent to the study protocol which was approved by the Ethic commission of the Medical University of Vienna (EK Nr. 571/2007).

All investigations were done using a 1.5T MRI (Siemens Avanto 1.5T; Siemens Erlangen, Germany).

The volunteers were placed in supine position and were asked to actively move their heads into maximum flexion and extension and were then supported with cushions to remain in the respective position during MRI data acquisition. T2-weighted median-sagittal slices showing the entire contours of the vertebral bodies C3 to C7 were used for biomechanical calculation of the respective CORs. Only datasets with no degenerative disc disease or similar degenerative changes were used for calculation.

### Prostheses

We selected 3 prosthesestypes with a considerably different biomechanical concept: one with a ball socket design and its COR below (Discover; DePuy Spine, Raynham, MA, USA); one with an inverse ball socket design allowing longitudinal translation and its COR above (Prestige STLP; Medtronic, Minneapolis, MN, USA); and one with 2 articulating surfaces and a flexible COR (Bryan; Medtronic, Minneapolis, MN, USA).

### Patients

Thirty patients (20 females, 10 males, age 34–59 years, mean 45 years) who received a cervical disc prosthesis in one level were included in the study, 10 patients who received a Bryan prosthesis, 8 of them at C5/6 and 2 of them at C6/7; 10 patients who received a Discover prosthesis, 1 of them at C4/5, 6 at C5/6 and 3 at C6/7; and 10 patients who received a Prestige STLP prosthesis, 6 of them at C5/6 and 4 at C6/7. Twenty-six patients were operated at the author’s institution and 4 patients were operated elsewhere; all of them for the generally accepted indications for arthroplasty. Surgery was performed through a standard anterior approach with micro-discectomy using the operating microscope. Correct implant position was verified intra-operatively with fluoroscopy.

The routinely taken pre- and post-operative cervical flexion/extension x-rays were collected from their files for biomechanical analysis. If the necessary landmarks for biomechanical calculation could not be determined at C6/7 because C7 was covered from the patients’ shoulders, data for C6/7 was spared from the respective dataset. Also, data was spared from further analysis when segmental ROM was below 2^o^ and therefore reliable calculation of the COR was not possible.

### Coordinate system

**Fig. 1:**
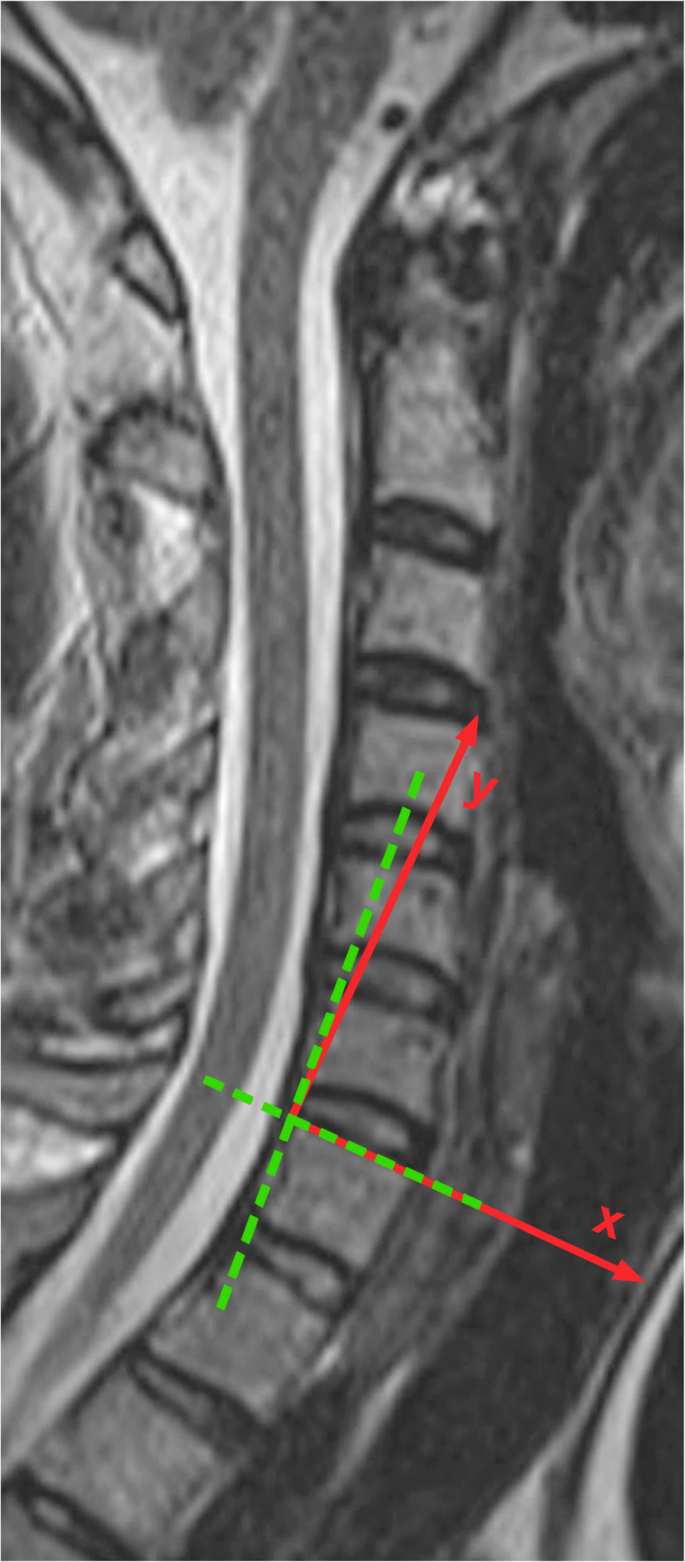
Definition of the coordinate system

The coordinate-system for motion analysis of flexion/extension was determined using a line through the most superior anterior and the most superior posterior point of the respective vertebral body. The cutting point with a second line through the most posterior inferior and the most posterior superior point of the respective vertebral body was defined as the center of the coordinate system with the *x*-axis passing through the most superior anterior point of the respective vertebral body, the *y*-axis directing cranially rectangular to the *x*-axis, and the *z*-axis rising orthogonally against the viewer (Fig. 1). MRI slices were obtained in 3 mm thickness with the same study protocol used for diagnostic MR-imaging of the cervical spine.

### Motion analysis

**Fig. 2:**
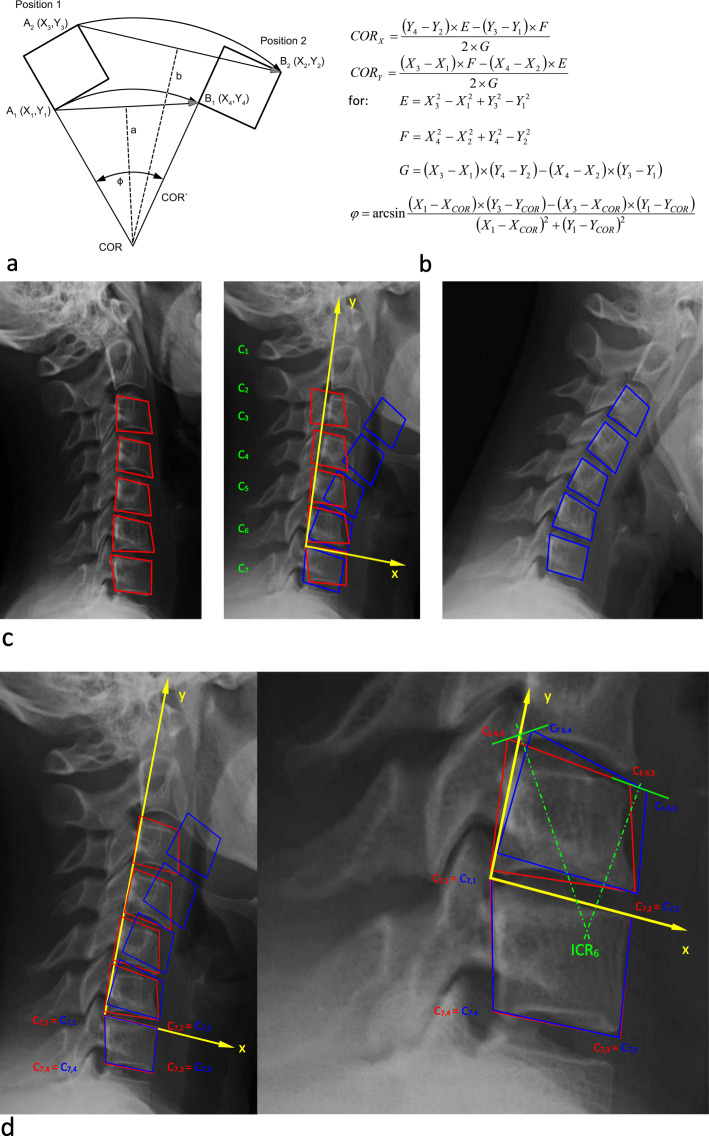
Determination of the COR. **a** Graphically illustrates the determination of the COR. **b** The respective mathematical algorithm. **c** An example how the vertebral bodies in lateral flexion/extension x-rays are covered with quadrangles, the respective quadrangles of the lower vertebral body are matched, and the quadrangles of the next cranial vertebral body allow graphical or mathematical determination of the COR. **d** An example how the COR C6/7 can be determined using this technique

The COR—or also described as instant center of rotation ICR in several studies—is commonly used for qualitative motion analysis and can describe how adjacent vertebral bodies move against each other [9]. Figure 2 illustrates the determination of the COR and shows an example how this was done with functional x-rays, for instance. Both the MRI pictures and the functional x-rays were manually digitized using AutoCAD® software (AutoCAD®, AUTODESK, San Rafael, CA, USA). The vertebral bodies were covered with a quadrangle to allow better overlay of the respective vertebral bodies and to use all four edge-points of the quadrangle for a more precise calculation of the COR. Coordinate calculation was done using Microsoft Excel® software (Microsoft Excel®, Microsoft, Redmont, WA, USA)

### COR

COR for flexion/extension was calculated for all healthy volunteers and all patients from C3/4 to C6/7 (if possible), and separately for the 3 respective prostheses subgroups; pre- and post-op CORs were compared with the coordinates found in healthy volunteers; thus, it was determined whether the COR changed its position toward or away from the COR-HV after insertion of the 3 different types of disc prostheses.

### Statistical analysis

The *t* test was used for determination of significance regarding the differences between the respective data-sets with a significance level of α = 0.05.

## Results

Data for C6/7 was spared from the datasets Bryan08—post-op; Discover09 pre- and post-op; Discover10 pre-op; Prestige05 pre-op because the necessary landmarks for biomechanical calculation could not be determined as C7 was covered from the patients’ shoulders. Also data was spared from analysis from the datasets Bryan05–C5/6 pre-op; Bryan08–C5/6 post-op because segmental ROM was below 2° and therefore reliable calculation of the COR was not possible. The post-op Prestige03-dataset was not suitable for COR analysis and was therefore completely excluded from further analysis.

### ROM healthy volunteers

Fifteen datasets were analyzed; the mean ROM for flexion/extension from C3 to C7 was 53.4° (SD 12.7). Table 1 shows the mean values for maximum flexion/extension of the respective motion segments

**Table 1:** ROM C3–C7 pre- and post-op for all operated patients and for the respective subgroups Bryan, Discover, and Prestige Prosthesis and ROM in healthy volunteers HV

ROM ° (SD)	HV	All patients		Bryan		Prestige		Discover	
		Pre-op	Post-op	Pre-op	Post-op	Pre-op	Post-op	Pre-op	Post-op
C3/4	11.4 (3.4)	8.5 (3.7)	11.3 (3.9)	8.7 (4.0)	10.6 (3.7)	8.6 (2.9)	11.9 (3.2)	8.2 (4.6)	11.4 (5.0)
C4/5	14.9 (4.8)	11.9 (4.7)	13.5 (4.3)	12.5 (5.5)	14.9 (3.8)	13.2 (3.7)	12.6 (3.2)	10.0 (4.5)	12.8 (5.5)
C5/6	12.7 (3.4)	10.1 (4.7)	11.6 (5.4)	10.4 (6.3)	11.3 (5.0)	9.9 (4.3)	12.4 (7.5)	9.9 (3.8)	11.2 (3.9)
C6/7	14.4 (5.8)	7.6 (4.1)	7.4 (4.1)	8.0 (4.7)	7.6 (4.5)	7.5 (4.9)	7.3 (5.0)	7.5 (2.5)	7.1 (3.1)
C3–C7	53.3 (12.7)	38.3 (11.7)	43.4 (8.9)	39.5 (15.3)	44.4 (9.1)	37.7 (8.2)	43.9 (6.8)	37.6 (11.1)	41.5 (11.0)

### ROM patients

Twenty-six datasets could be analyzed for pre- and post-operative ROM from C3 to C7; 10 from the Bryan subgroup, 8 from the Discover subgroup, and 8 from the Prestige subgroup. The mean ROM for flexion/extension from C3 to C7 was pre-operatively 38.3° (SD 11.7) and post-operatively 43.4° (SD 8.9). The mean ROM of the respective segments both for all patients and for the 3 subgroups are shown in Table 1.

### COR for maximum flexion/extension healthy volunteers

**Fig. 3:**
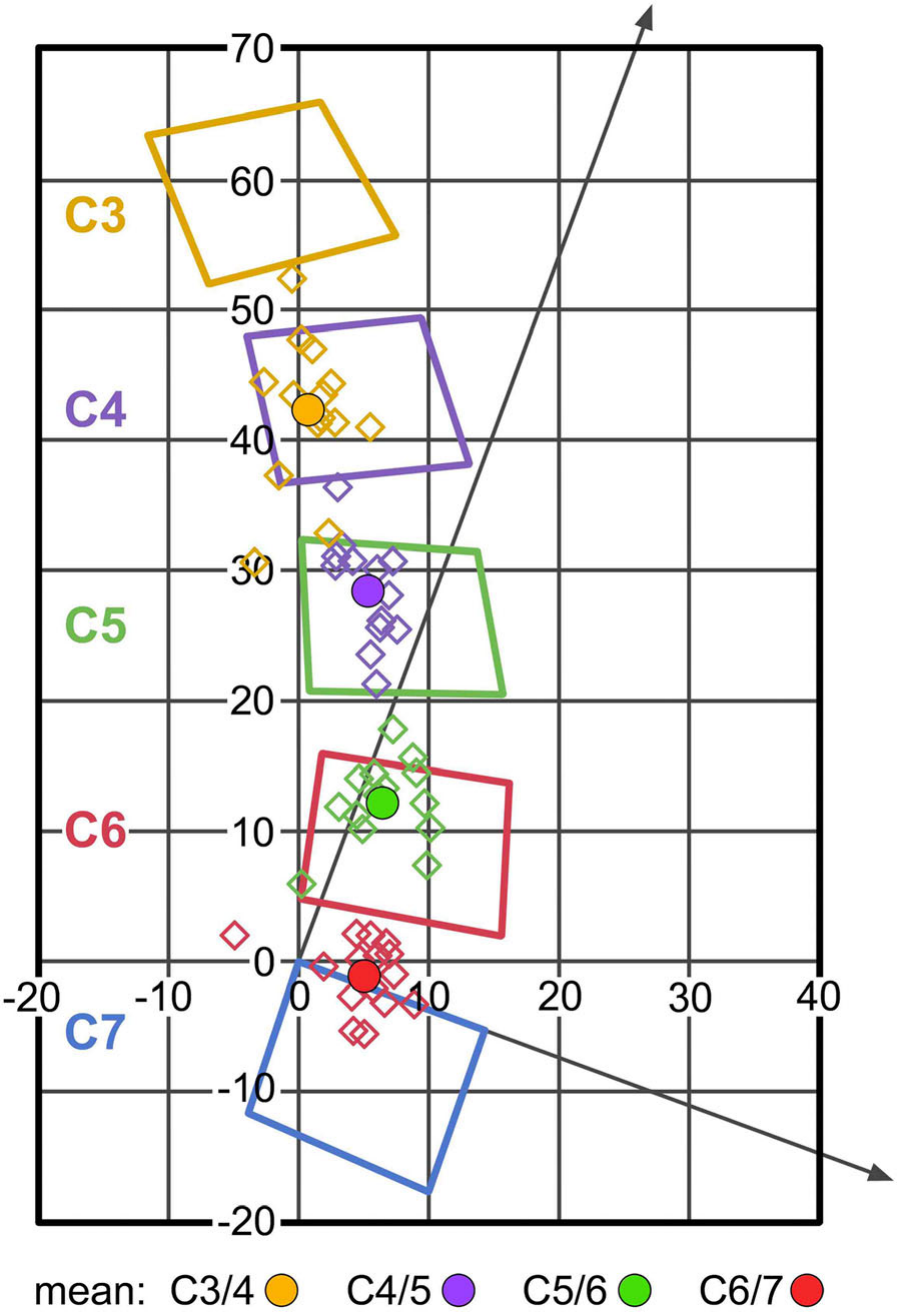
Position of COR for maximum flexion/extension in healthy volunteers

The following coordinates (mean, SD) were found for flexion/extension: C3/4: *x*4.8/*y*-5.8 (2. /5.6); C4/5: *x*4.8/*y*-3.8 (2.3/3.7); C5/6: *x*4.8/*y*-4.0 (2.8/3.1); C6/7: *x*4.9/*y*-1.1 (3.2/2.6) (Fig. 3).

### COR for maximum flexion/extension all patients

**Fig. 4:**
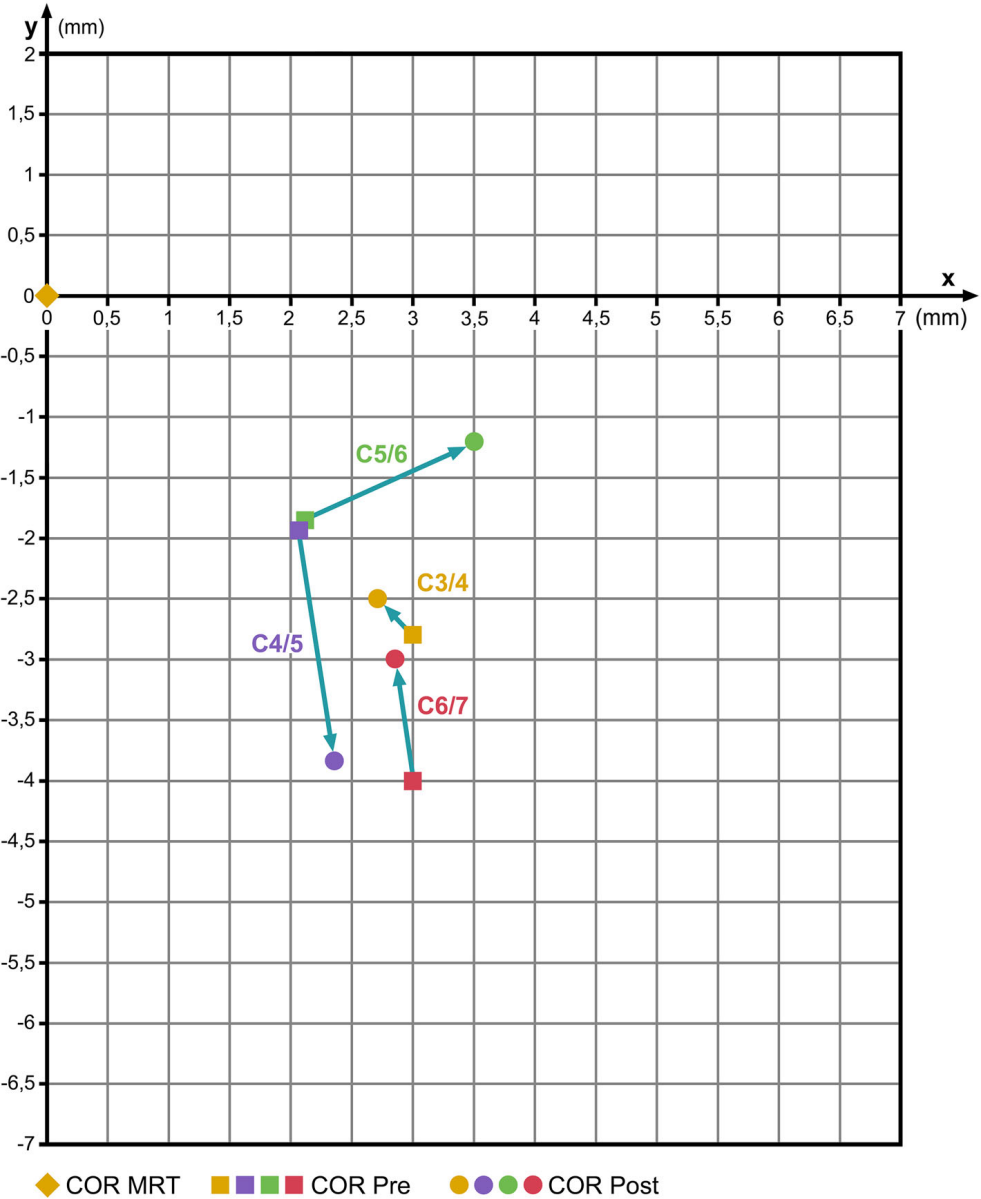
Change of COR after insertion of a disc prosthesis irrespective of the prosthesis type. Coordinate origin 0/0 represents the respective COR in healthy volunteers

Table 2 shows the *x*- and *y*-coordinates of the COR from all patients and levels. Table 3 compares the pre- and post-operative COR-coordinates (mean values) from all patients—irrespective of the prosthesis type and the operated level—with the COR-HV. It shows that the pre-operative *x*-coordinates of the patients differ considerably in all levels from the COR-HV, and that the pre-operative COR for flexion/extension is located more anteriorly than in healthy subjects. Post-operatively, the COR for C3/4 remains nearly unchanged. The CORs for C4/5 and C5/6 shift away from the normal COR-HV (C4/5 inferiorly, C5/6 anteriorly). For the COR C6/7, the *x*-coordinates remain nearly unchanged and the *y*-coordinates mildly shift toward the normal COR-HV (Fig. 4).

**Table 2:** COR pre- and post-op: all patients and all levels

	Level	C6/C7				C5/C6				C4/C5				C3/C4			
		***x***		***y***		***x***		***y***		***x***		***y***		***x***		***y***	
		Pre	Post	Pre	Post	Pre	Post	Pre	Post	Pre	Post	Pre	Post	Pre	Post	Pre	Post
**Bryan01**	C6/C7	6.1	17.4	− 2	− 4.5	5.9	4.6	− 6	− 6.5	3.8	9.6	− 6.7	− 7.8	7.3	9.3	− 6.2	− 10.7
**Bryan02**	C5/C6	10.8	5.6	− 3.2	− 0.8	9.8	12.3	− 8.1	− 8.7	3.9	8.4	− 7.7	− 5.1	8.8	6.3	− 5	− 9.3
**Bryan03**	C5/C6	3.3	1.6	− 8.9	− 6.7	6.6	9.6	− 9.7	− 10.6	3	4.7	− 7.7	− 7.9	4.3	9.5	− 7	− 9.4
**Bryan04**	C5/C6	8.3	11.1	2.1	3.6	6.1	8.2	− 17	− 5.5	10.7	4.1	4.7	− 9.2	10.8	3.5	12	− 8.7
**Bryan05**	C6/C7	14.9	6.5	− 3.5	− 9.2		7.7		− 6.7	6.9	5.2	− 23.5	− 11.7	27.2	5.7	− 14.2	− 12.3
**Bryan06**	C5/C6	7.9	− 5.1	0.2	− 0.1	7.3	7.8	− 12.3	− 12.3	7.9	7.5	− 7.2	− 7.2	8.8	7.3	− 15.6	− 12.1
**Bryan07**	C5/C6	3.7	− 2.1	− 12.1	1	8.1	9.2	− 5.1	− 3.8	8.8	8.9	− 6.5	− 10.9	10.6	12.7	− 10.9	− 10.9
**Bryan08**	C5/C6	10.7		− 35.7		− 8.4		− 2.4		4.3	− 0.5	− 5.4	− 11	11.5	6.1	− 5.7	− 13.8
**Bryan09**	C5/C6	4.9	4.1	− 4	− 0.7	8.7	14.5	− 6.8	8.5	10.9	8.2	− 7.5	− 7.3	8.2	10.4	− 4.3	− 5.9
**Bryan10**	C5/C6	6.6	12.5	4.2	2	2	2.5	− 5.6	− 5.2	3.2	8.7	− 0.6	− 0.8	1.7	2.8	− 16	23.3
**Discover01**	C6/C7	5.9	8.6	0.1	− 1.4	6.1	7.1	− 5.7	− 1.2	2.5	7.4	− 8.2	− 1.7	7.7	6.6	− 4.1	2.8
**Discover02**	C5/C6	7.3	7.6	3.2	− 4.6	4.4	10.3	− 5.2	− 13.2	1.9	7.5	− 10.3	− 9.8	3.3	8.6	− 12.3	− 10.8
**Discover03**	C5/C6	13.8	13.9	− 1.5	9.4	11.9	10.4	7.4	2.1	7.2	6.4	− 2.9	− 1.3	12.5	15.7	− 7	− 8.6
**Discover04**	C5/C6	9.1	6.7	− 5.4	− 7.4	7.5	6.5	− 8.1	− 9.1	5.1	7	− 2.5	− 7.6	3	7.7	− 16.1	− 11.2
**Discover05**	C6/C7	9.5	8.5	− 0.7	− 7.1	8.5	15.2	− 5.5	− 11	7.9	9	− 8.8	− 11.5	7.5	15.6	− 7.3	− 21.7
**Discover06**	C5/C6	13.7	15.5	− 1.5	− 35.7	4.8	8.4	− 5.9	− 7.6	7.3	8.1	− 5.4	− 6.1	8.8	7.6	− 5.1	− 6.6
**Discover07**	C5/C6	7.6	7.4	− 7.1	− 20.8	9.7	12	− 10.1	− 6.8	9.2	27.8	− 5	− 12.9	3.1	10.9	− 10.4	− 7.9
**Discover08**	C4/C5	3.7	11.2	− 3	3.6	6.6	7.6	− 5.3	− 3.9	4	8.3	− 8.1	− 9.6	3	10.8	− 14.1	− 10.6
**Discover09**	C5/C6					8.5	6.3	− 6.5	− 5.8	17.6	6.1	17.8	− 6.8	6.5	8.7	− 16.4	− 6.3
**Discover10**	C6/C7	3.2		− 0.7		9.3	15	− 1.1	− 8.5	7.5	2.3	− 5.2	− 6.5	2.2	5.9	− 4.9	− 5.8
**Prestige01**	C6/C7	13.4	− 2.1	− 14.1	− 7.9	8.5	8.1	− 5	− 4.7	9.9	6.4	− 8	− 5.1	12.2	5.5	− 7.6	− 10.4
**Prestige02**	C6/C7	8.5	4.8	− 3.8	− 5.1	7.2	8.2	− 8.5	− 8.6	13	9.7	− 2.7	− 8.1	8.3	8.5	− 5.9	− 6.9
**Prestige03**	C5/C6					5.3		− 10.5		9.3		− 6.9		12.8		− 12.3	
**Prestige04**	C6/C7	6.5	6.6	− 0.3	− 2.1	20.2	11.8	6.4	− 2.9	8	5.3	− 7.9	− 8.9	4	6.7	− 9	− 5.3
**Prestige05**	C5/C6	7.1		0.5		6.1	8.3	− 2.3	6.6	4.2	9.3	− 8.4	− 8.6	5	14.7	− 12.3	− 8.8
**Prestige06**	C5/C6	4.9	− 2	− 32.8	− 37.7	7.5	0.2	− 8.5	− 9.6	10.1	6	− 10	− 12.7	8.7	1.7	− 8.3	− 11.9
**Prestige07**	C5/C6	− 2.7	10.8	− 1.9	− 7	6	6.8	− 7.9	− 8.1	2.9	1.1	− 5.5	− 5.6	4.8	− 0.2	− 8.9	− 13.6
**Prestige08**	C5/C6	2.8	9	− 1.3	6.5	7.7	6.6	− 5.4	− 4.3	2.6	2.5	− 4.9	− 8.7	4.7	1.8	− 11.6	− 5.8
**Prestige09**	C5/C6	8.7	7	− 1.3	0	6.2	5.5	− 4.6	1.9	7.5	1.5	− 3.5	− 3.5	4.8	1.2	− 3.4	− 8
**Prestige10**	C6/C7	19.7	27.6	− 7.4	30.2	1.6	2.2	− 5.2	0.3	4.4	8.5	− 5	− 7.7	10.6	5.5	− 6.9	− 14.1
**Mean**		7.9	7.7	− 5.1	− 4.1	6.9	8.3	− 5.9	− 5.2	6.9	7.1	− 5.7	− 7.6	7.8	7.5	− 8.6	− 8.3
**Median**		7.5	7.4	− 2.0	− 2.1	7.2	8.2	− 5.7	− 6.2	7.3	7.4	− 6.6	− 7.8	7.6	7.3	− 8.0	− 9.3
**SD**		4.5	7.0	9.2	13.2	4.4	3.6	4.7	5.3	3.6	4.8	6.2	3.2	4.9	4.1	5.6	7.4
***p*** **value**		0.928		0.757		0.187		0.604		0.844		0.130		0.820		0.888	

*x*- and *y*-coordinates in millimeters

**Table 3:** COR mean values pre- and post-op: all patients—irrespective of implanted prosthesis—compared with COR in healthy volunteers (COR-HV)

Segment		COR-HV		COR pre-op		COR post-op	
		*x*	*y*	*x*	*y*	*x*	*y*
C6/7	Mean	4.9	− 1.1	7.9	− 5.1	7.7	− 4.1
	SD	3.2	2.6	4.5	9.2	7.0	13.2
	*p*			0.029	0.115	0.146	0.397
C5/6	Mean	4.8	− 4.0	6.9	− 5.9	8.3	− 5.2
	SD	2.8	3.1	4.4	4.7	3.6	5.3
	*p*			0.097	0.179	0.002	0.443
C4/5	Mean	4.8	− 3.8	6.9	− 5.7	7.1	− 7.6
	SD	2.3	3.7	3.6	6.2	4.8	3.2
	*p*			0.055	0.305	0.099	0.001
C3/4	Mean	4.8	− 5.8	7.8	− 8.6	7.5	− 8.3
	SD	2.3	5.6	4.9	5.6	4.1	7.4
	*p*			0.035	0.123	0.026	0.248

### Bryan subgroup

**Fig. 5:**
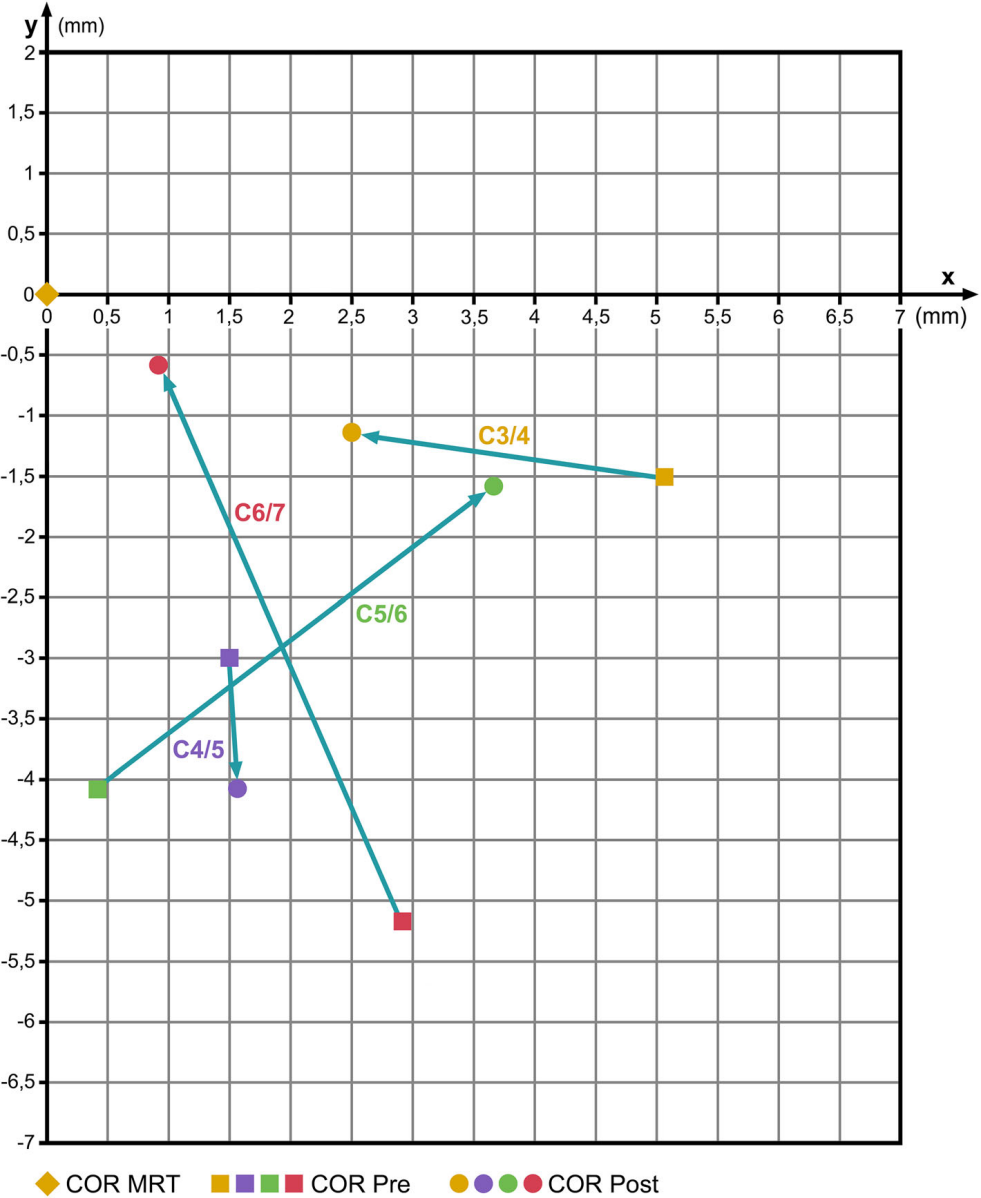
Change of COR after insertion of a Bryan disc prosthesis. Coordinate-origin 0/0 represents the respective COR in healthy volunteers

Table 4 summarizes the pre- and post-operative COR-coordinates (mean values) from the Bryan-subgroup compared with the COR-HV; the COR for C3/4 is shifted toward the normal COR-HV. The COR for C4/5 nearly remains unchanged. For C5/6, the *x*-coordinates of the COR are shifted away from normal and the *y*-coordinates are shifted toward the normal COR-HV. For C6/7 the COR is shifted toward the normal COR-HV (Fig. 5).

**Table 4:** COR mean values pre- and post-op after implantation of a Bryan prosthesis compared with COR in healthy volunteers (COR-HV)

Segment		COR-HV		COR pre-op		COR post-op	
		*x*	*y*	*x*	*y*	*x*	*y*
C6/7	Mean	4.9	− 1.1	7.7	− 6.3	5.7	− 1.7
	SD	3.2	2.6	3.6	11.4	7.2	4.2
	*p*			0.049	0.101	0.684	0.676
C5/6	Mean	4.8	− 4.0	5.1	− 8.1	8.5	− 5.6
	SD	2.8	3.1	5.5	4.4	3.6	6.0
	*p*			0.832	0.014	0.010	0.389
C4/5	Mean	4.8	− 3.8	6.3	− 6.8	6.5	− 7.9
	SD	2.3	3.7	3.1	7.1	3.1	3.2
	*p*			0.173	0.182	0.139	0.010
C3/4	Mean	4.8	− 5.8	9.9	− 7.3	7.4	− 7.0
	SD	2.3	5.6	6.8	8.1	3.1	10.9
	*p*			0.012	0.587	0.028	0.720

### Discover subgroup

**Fig. 6:**
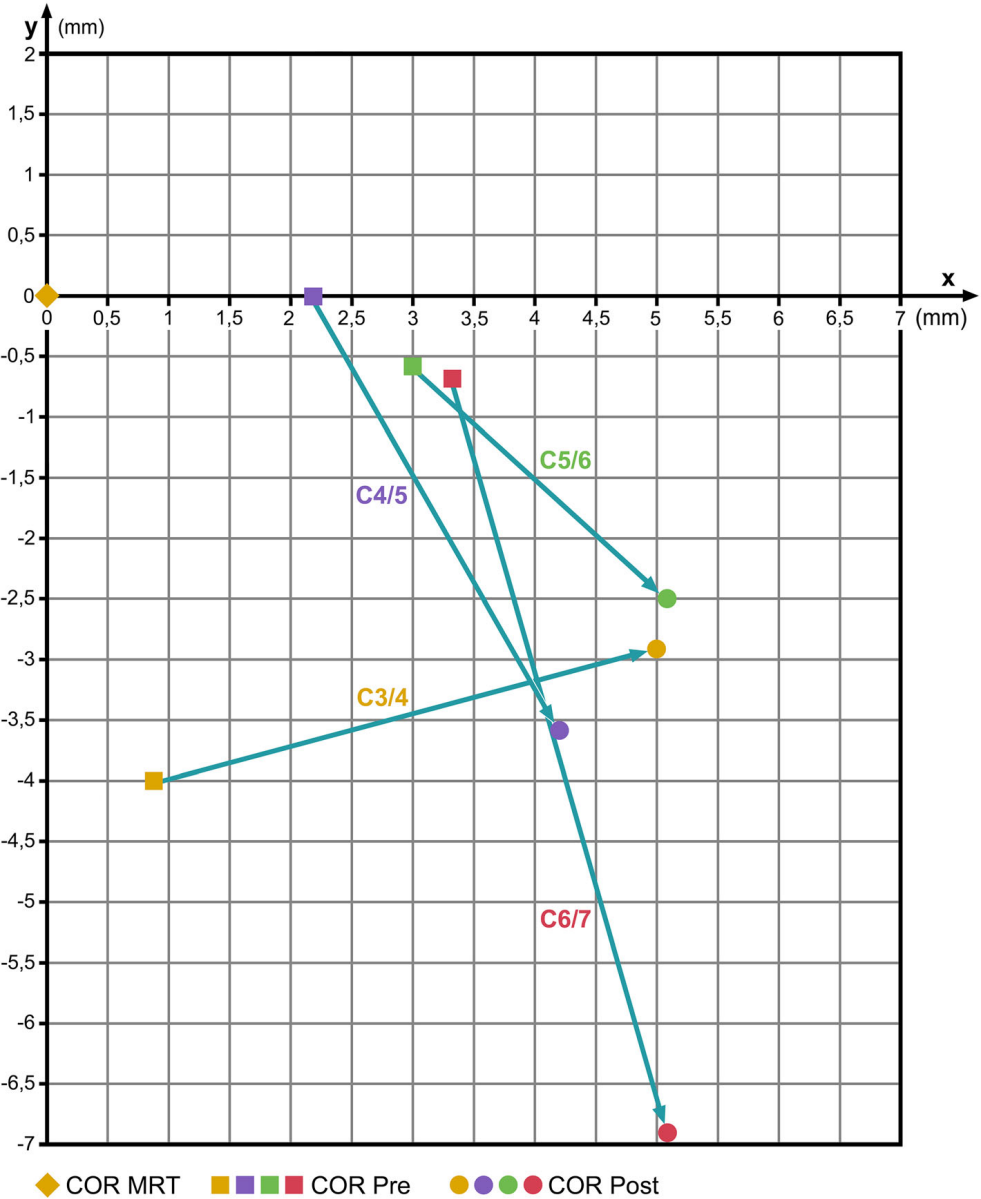
Change of COR after insertion of a Discover disc prosthesis. Coordinate-origin 0/0 represents the respective COR in healthy volunteers

Table 5 summarizes the pre- and post-operative COR-coordinates (mean values) from the Discover-subgroup compared with the COR-HV; in C3/4, the *x*-coordinates of the COR are considerably shifted away from normal, and the *y*-coordinates are mildly toward the normal COR-HV. However, in C4/5, C5/6, and C6/7, the COR is considerably shifted away from the normal COR-HV both for the *x*- and the *y*-coordinates (Fig. 6).

**Table 5:** COR mean values pre- and post-op after implantation of a Discover prosthesis compared with COR in healthy volunteers (COR-HV)

Segment		COR-HV		COR pre-op		COR post-op	
		*x*	*y*	*x*	*y*	*x*	*y*
C6/7	Mean	4.9	− 1.1	8.2	− 1.8	9.9	− 8.0
	SD	3.2	2.6	3.8	3.0	3.3	14.3
	*p*			0.031	0.542	0.002	0.079
C5/6	Mean	4.8	− 4.0	7.7	− 4.6	9.9	− 6.5
	SD	2.8	3.1	2.3	4.8	3.3	4.6
	*p*			0.011	0.721	0.000	0.118
C4/5	Mean	4.8	− 3.8	7.0	− 3.9	9.0	− 7.4
	SD	2.3	3.7	4.4	8.0	6.9	3.8
	*p*			0.117	0.988	0.039	0.030
C3/4	Mean	4.8	− 5.8	5.8	− 9.8	9.8	− 8.7
	SD	2.3	5.6	3.4	4.7	3.5	6.1
	*p*			0.419	0.076	0.000	0.234

### Prestige subgroup

**Fig. 7:**
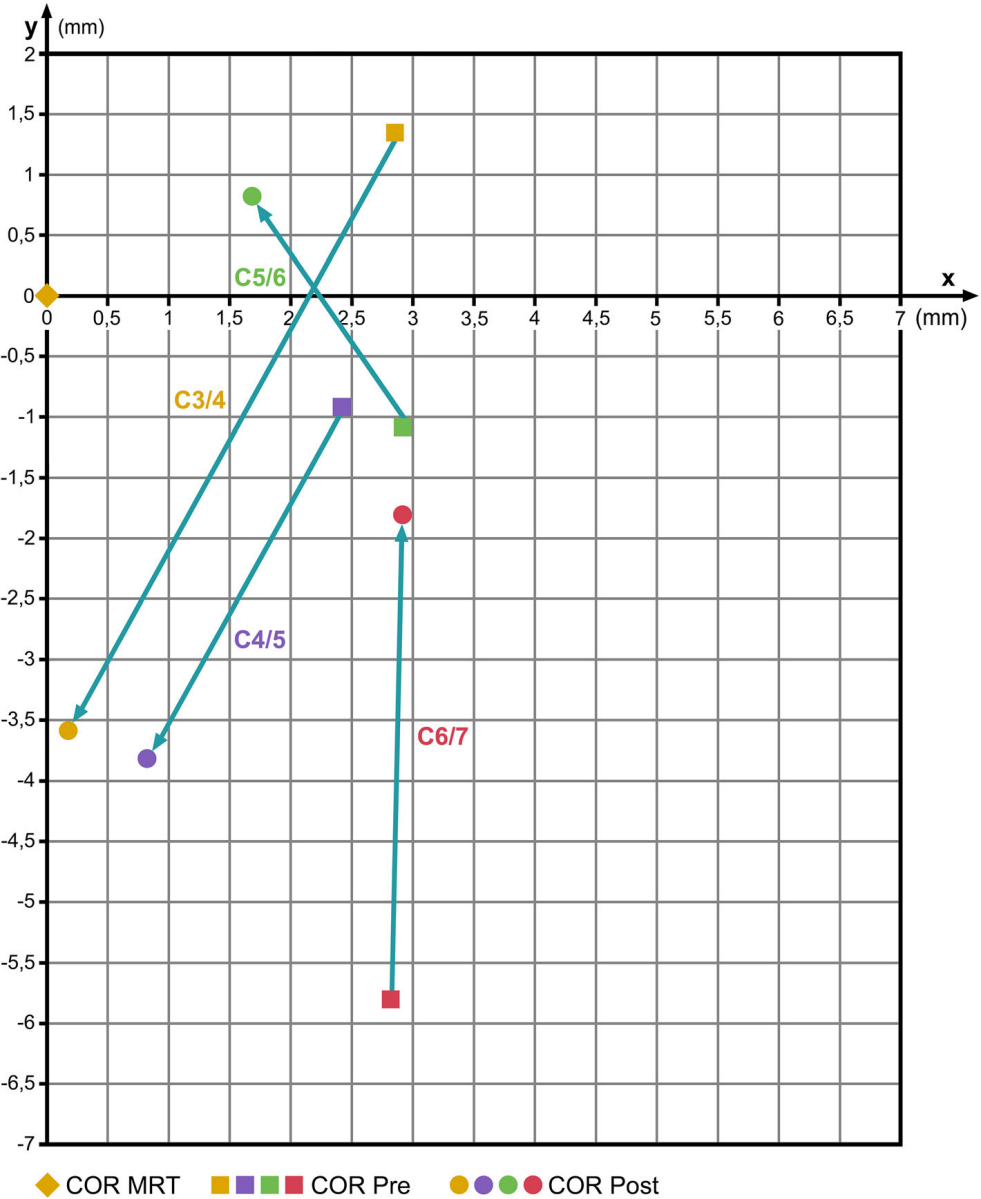
Change of COR after insertion of a Prestige disc prosthesis. Coordinate-origin 0/0 represents the respective COR in healthy volunteers

Table 6 summarizes the pre- and post-operative COR-coordinates (mean values) from the Prestige subgroup compared with the COR-HV; for the CORs for C3/4 and C4/5, the *x*-coordinates are shifted near to normal, but the *y*-coordinates are shifted considerably away from the normal COR-HV. In C5/6, the *x*-coordinates are shifted mildly toward normal and the *y*-coordinates switch from negative to positive values with a mild improvement toward the normal COR-HV. In C6/7, the *x*-coordinates remain nearly unchanged, and the *y*-coordinates are shifted toward the normal COR-HV (Fig. 7).

**Table 6:** COR mean values pre- and post-op after implantation of a Prestige prosthesis compared with COR in healthy volunteers (COR-HV)

Segment		COR-HV		COR pre-op		COR post-op	
		*x*	*y*	*x*	*y*	*x*	*y*
C6/7	Mean	4.9	− 1.1	7.7	− 6.9	7.7	− 2.9
	SD	3.2	2.6	6.3	10.7	9.3	18.7
	*p*			0.163	0.054	0.289	0.718
C5/6	Mean	4.8	− 4.0	7.6	− 5.2	6.4	− 3.3
	SD	2.8	3.1	4.8	4.7	3.5	5.4
	*p*			0.072	0.479	0.216	0.660
C4/5	Mean	4.8	− 3.8	7.2	− 4.7	5.6	− 7.7
	SD	2.3	3.7	3.5	2.3	3.3	2.7
	*p*			0.052	0.078	0.511	0.014
C3/4	Mean	4.8	− 5.8	7.6	− 4.5	5.0	− 9.4
	SD	2.3	5.6	3.4	2.9	4.6	3.3
	*p*			0.022	0.153	0.881	0.090

These findings claim the following results:

1. The pre-operative COR is not congruent with the COR found in healthy subjects; once a disc herniations occurs, the motion pattern changes, and the pre-op COR should not be used as reference anymore.
2. The post-op position of the COR considerably varies depending on the biomechanical concept of the prosthesis: devices with a ball socket design (fixed COR) can shift the COR considerably away from the physiological COR; prostheses with inverse ball socket design and potential for longitudinal translation can shift the *x*-coordinates moderately toward normal, but the *y*-coordinates can be shifted considerably off from the COR-HV; prostheses with variable COR can considerably improve the position of the COR toward normal at least in certain motion segments.
3. The post-op shift of the COR was noted in all investigated levels irrespective of the operated level. This demonstrates that biomechanical changes in one motion segment can cause a cascade of compensatory effects in all other levels.
4. As long as it is not scientifically proven that prosthesis design has no impact on clinical outcome, surgeons should choose devices with a more sophisticated biomechanical concept considering that the COR varies between segments and also changes its position during motion.

## Discussion

A great variety of biomechanical studies have been published about cervical arthroplasty. Most studies focus on the ROM or on changes in disc height and facet translation compared with normal individuals and with patients after ACDF or on clinical results [10–22]. Several studies also investigate qualitative motion of disc prostheses [1–9], but we did not find studies investigating how the COR after insertion of a prosthesis compares with healthy individuals, which is important because our data shows that the COR in patients with disc herniations is not at a physiological position anymore and therefore the pre-op COR cannot be used as reference:

Anderson et al. undertook a meta-analysis on kinematics of the cervical adjacent segments after disc arthroplasty compared with anterior discectomy and fusion [10]. Twelve papers were identified to meet the inclusion criteria, but only 2 of them—Park et al. [5] and Powell et al. [7]—investigated COR. The Powell study compared COR after 22 Bryan prostheses with 26 patients after ACDF. At the index level, the COR shifted more posterior (0.3 mm) and cephalad (4.9 mm) post-operatively. At the adjacent level above, COR was significantly posterior compared with fusion. There was no significant difference at the level below fusion. The study published by Park et al. investigated 272 patients after arthroplasty with a PCM cervical artificial disc; at the index-level, COR-x was found 0.8 mm posterior to the disc center before surgery and 0.2 mm anterior to the center at 12 months after TDR. COR-y was 2.5 mm below the endplate before and 4.0 mm at 12 months after surgery. COR at the adjacent levels was unaltered in this study by fusion or arthroplasty. Comparing these 2 studies reveals that different biomechanical prostheses concepts also influence the post-op position of the COR differently; however, other than in our study, none of these 2 studies provides data whether the change of the COR represents an improvement toward physiological values at the respective levels or rather a further shift out of the physiological range of a normal COR.

Liu et al. presented an in vivo study investigating the inter-segmental ROMs and introducing a mathematical model calculating contact forces of normal, fused, and post-arthroplasty cervical spines; they concluded that arthroplasty can preserve motion and force patterns of the normal cervical spine, but no data are presented on COR [4].

Pickett et al. investigated the COR in 20 patients after receiving a Bryan artificial disc [6]. It was found that the COR did not change significantly at the index level or any other level after surgery. They state that the Bryan device is able to provide a clinically adequate range of COR; but no comparison with normal values is presented. In our study, the COR after inserting a Bryan prosthesis was found more close to the COR-HV than with the other devices.

Rousseau et al. compared the kinematics of the Prestige LP prosthesis with the ProDsic-C prosthesis in an in vivo study [8]. The COR for the Prestige LP was found above the disc level and the COR for the ProDisc C below it. Only average values for both CORs are presented. They conclude that arthroplasty devices using a ball socket design influence inter-vertebral kinematics for flexion/extension. We found similar biomechanical properties for the Prestige LP and the Discover (which is in its biomechanical design very similar to the ProDisc C), and we believe this reflects typical motion patterns for devices with the COR above disc level (Prestige) or below disc level (Discover, ProDisc C).

Koller et al. investigated biomechanical changes after insertion of a Discover prosthesis in 19 patients [1]. They found that COR-x shifted anteriorly outside of normal limits in approximately 50% of their patients. The shell angle and the position of the prostheses significantly correlated with the position of the COR-x and the COR-y. However, other than in our study, their COR data were pooled and overlaid on the C5/6 level. Still, their findings compare well with our data from the Discover patients showing worsening of the post-op COR due to the fixed ball socket design.

Kowalczyk et al. presented an in vivo kinematic study comparing the Bryan-, ProDisc C, and the Prestige LP prostheses and their impact on the sagittal balance and segmental kinematics of the cervical spine [2]. In their study, the Bryan disc did not change the COR-x or COR-y significantly; in the ProDisc-C group, the COR-x was shifted anteriorly, and in the Prestige LP group, the COR-y was shifted superiorly. This is similar to our findings for these 3 different biomechanical concepts; however, other than in our study, no data on how these findings compare to normal individuals are presented.

So, the findings from most of these studies support our claim that the biomechanical design of a prosthesis has an impact on the post-op motion pattern, and especially simple ball socket devices can make things even worse. Our study adds additional information how the post-op COR after inserting the different devices compares to healthy individuals. We believe this is important, because a disc prosthesis can only claim to resemble natural motion when the post-op motion pattern of the operated segment compares well to healthy subjects rather than to the motion pattern of a segment affected by a damaged disc.

Regarding clinical outcome, the literature does not support the wide-spread opinion that prosthesis-design does not matter: Upadhyaya et al. [22] published an analysis of 3 randomized multicenter US FDA investigational device exemption cervical arthroplasty trials (Heller et al. [14]: Bryan; Mummaneni et al. [18]: Prestige; Murrey et al. [19]: ProDisc C). Similar to our study, the prostheses in these trials represent the same 3 different biomechanical concepts: variable COR (Bryan), COR for flexion/extension above the disc level with longitudinal translation (Prestige), and fixed COR below the disc level and ball socket design (ProDisc C), which is very similar to the design of the Discover prosthesis which was investigated in our study. The improvement for the NDI in these studies was best for the Bryan device, followed by Prestige and ProDisc C. The improvement for neck pain frequency and intensity was best for the Bryan, followed by Prestige and ProDisc C. Neurological success was best with the Prestige prosthesis, followed by Bryan and ProDisc C. These findings support our opinion that a more flexible biomechanical prosthesis design can contribute to better clinical results. The ranking for NDI improvement in this study exactly reflects our findings for improvement of post-op COR.

However, a randomized study directly comparing clinical outcome for these devices is still lacking, and the papers presenting the 10-year results for these 3 devices are hardly comparable: regarding VAS neck/arm, for instance, for the BRYAN prothesis improvement of Δ54.3/Δ58.1 (75.4–20.9/71.2–14.1) is reported [23]; for PorDisc C Δ45/Δ42 (64–19/63–21) [24], but the considerable pre-op VAS-difference between BRYAN and ProDisc C allows no valid conclusion; and for the Prestige LP, VAS improvement is given as a percentage (60.7%/59.6%) [25] and therefore is not comparable either. A multi-center study relating clinical outcome to the biomechanical concepts of the respective prostheses would be highly desirable.

Staudt published a review on a variety of first- and second-generation cervical disc prosthesis and concludes that knowledge of implant design and design-specific advantages and disadvantages will become increasingly important to guide surgeon decision-making [26]. We completely share the opinion in this review, and we hope that our study can also contribute to decision-making in clinical practice by showing that a flexible biomechanical prosthesis-design can lead to better post-op qualitative motion.

Skeppholm reported a higher re-operation rate following cervical arthroplasty compared to ACDF; in this study, 151 patients received a Discover disc (DePuy Spine) and 21 a Prestige LP disc (Medtronic) compared to 504 patients with ACDF. The most common reason for re-operation in the arthroplasty group was implant migration or instability. Unfortunately, no data is given whether re-operation rate could be related to implant design [27], probably because of the significantly different cohorts (151 Discover/21 Prestige prostheses), but the question how implant migration in this study compares to the biomechanical findings for the same devices in our study would be of high interest.

Ryu et al. [28] investigated radiological changes of the operated and adjacent segments following cervical arthroplasty after a minimum 24-month follow-up: 19 patients with Bryan prosthesis were compared with 17 patients with ProDisc C prosthesis: progression of radiological degeneration at the index level was seen in 1 Bryan patient and in 6 ProDisc C patients. This is remarkable with respect to our findings, and the question arises whether the humble biomechanical concept of a fixed ball socket design not only influences qualitative motion to a worse pattern as is showed in our study, but also triggers further radiological degeneration.

Limitations of our study mainly arise from the small number of datasets, and therefore strong conclusions may not be drawn; we hope our findings will encourage others to initiate further studies with larger cohorts to define more precisely the biomechanical differences between healthy individuals and patients being candidates for cervical disc surgery.

Also, our patient data were collected retrospectively, but we believe this has little or no influence on determination of the COR.

Limitations also arise from the technique how the respective coordinates were determined: the inter-individual differences in the size of the vertebral bodies were not reflected when giving the coordinates of the COR in millimeters and not as a percentage of the vertebral bodies’ diameter. However, the error resulting from this limitation is expected below 1 mm and should not put our results into question.

Further, COR-HV was determined from MRI; there are techniques described in the literature that are more precise, like stereoradiography plus 3D-CT; however, this leads to radiation exposure of approx. 4MSV [29] which is a high burden for healthy volunteers. Also, differences in the motion pattern may occur whether the cervical spine is investigated in supine position in MRI compared to fluoroscopy where patients are in upright weight-bearing position with. But nevertheless, at least in Europe, one would hardly get permission from the Ethics Committee for a study design exposing healthy volunteers to such a radiation dose. We are aware that for these reasons perfect consistency between the MRI- and the fluoroscopic image coordinates cannot be achieved, but even taking into account such small error, we believe that the considerable differences we found for the COR-HV compared to the pre-op COR allow to claim that the COR in patients with disc herniations is not anymore where it is found in healthy subjects.

Even taking in account these limitations, we believe that our work shows how arthroplasty devices with different biomechanical concepts can influence post-operative cervical spine motion in a considerably different manner. Considering that flexion/extension is not a simple circular motion but has a variable COR even during motion, it must be concluded that disc prostheses with a simple ball socket design and fixed COR are not suitable to provide a physiological motion pattern for all cervical segments, and that devices with a more variable COR better contribute to re-establishing a more normal motion pattern. We hope that our study delivers useful new information for the daily practice of spine surgeons and will encourage anyone who is involved in cervical arthroplasty to look very closely on the biomechanical properties of the devices they chose.

## Conclusions

Even if the small cohorts in our study do not allow strong conclusions, it seems that once a disc herniations occurs, the motion pattern changes, and the COR is not anymore where it is in healthy individuals. If so, the pre-op COR cannot be used as a reference when discussing whether a disc prosthesis is resembling natural motion.

The biomechanical prosthesis-design considerably influences the post-op position of the COR: simple ball socket devices can shift the already abnormal COR to an even more unphysiological position; devices with a more flexible biomechanical design can contribute to normalize the coordinates of the COR.

An unphysiological post-op COR can cause a cascade of compensatory effects in all other levels.

Because there is no scientific evidence that prosthesis design does not influence clinical outcome, the biomechanical design should be taken into account when choosing an arthroplasty device, and only prostheses with flexible biomechanical properties should be used in clinical practice.

## Data Availability

The datasets used and/or analyzed during the current study (MRI pictures and X-rays used for biomechanical calculations) are available from the corresponding author on reasonable request.

## Abbreviations

ACDF: Anterior cervical discectomy and fusion
COR: Center of rotation
COR-HV: Center of Rotation in Healthy Volunteers
NDI: Neck Disability Index
ROM: Range of motion
