# Correction to: In cervical arthroplasty, only prosthesis with flexible biomechanical properties should be used for achieving a near-physiological motion pattern

**DOI:** 10.1186/s13018-020-02110-w

**Published:** 2020-11-26

**Authors:** Manfred Muhlbauer, Ernst Tomasch, Wolfgang Sinz, Siegfried Trattnig, Hermann Steffan

**Affiliations:** 1 Neurosurgical Department, Donauspital SMZ-Ost, Langobardenstrasse 122, 1220 Vienna, Austria; 2 Vehicle Safety Institute, Graz University of Technology, Graz, Austria; 3 High Field MR Center, Department of Biomedical Imaging and Image-guided Therapy, Medical University of Vienna, Vienna, Austria

**Correction to: J Orthop Surg Res (2020) 15: 391**

**https://doi.org/10.1186/s13018-020-01908-y**

Following publication of the original article [1], the authors identified an error in the author name of Ernst Tomasch.

The incorrect author name is: Ernst Thomasch

The correct author name is: Ernst Tomasch

The original article has been corrected.
